# A Wireless Monitoring System for Cracks on the Surface of Reactor Containment Buildings

**DOI:** 10.3390/s16060883

**Published:** 2016-06-14

**Authors:** Jianguo Zhou, Yaming Xu, Tao Zhang

**Affiliations:** 1 School of Geodesy and Geomatics, Wuhan University, Wuhan 430079, China; ymxu@sgg.whu.edu.cn (Y.X.); tzhang@sgg.whu.edu.cn (T.Z.); 2 Key Laboratory of Precise Engineering and Industry Surveying, National Administration of Surveying, Mapping and Geoinformation, Wuhan 430079, China

**Keywords:** structural health monitoring, wireless sensor networks, containment crack monitoring, real-life deployment

## Abstract

Structural health monitoring with wireless sensor networks has been increasingly popular in recent years because of the convenience. In this paper, a real-time monitoring system for cracks on the surface of reactor containment buildings is presented. Customized wireless sensor networks platforms are designed and implemented with sensors especially for crack monitoring, which include crackmeters and temperature detectors. Software protocols like route discovery, time synchronization and data transfer are developed to satisfy the requirements of the monitoring system and stay simple at the same time. Simulation tests have been made to evaluate the performance of the system before full scale deployment. The real-life deployment of the crack monitoring system is carried out on the surface of reactor containment building in Daya Bay Nuclear Power Station during the in-service pressure test with 30 wireless sensor nodes.

## 1. Introduction

Civil infrastructure usually plays an important role in society’s prosperity [1]. The safety of these structures during their normal operations cannot be neglected as any health problem as a result from them may cause severe life and property loss, which has happened many times in the past. Due to aging, loadings, environmental conditions and extreme events like earthquakes, structures may deteriorate in their performance slowly, or be damaged severely. Then, structural health monitoring (SHM) [2], which is defined as detecting changes to the material or geometric properties of structures, becomes essential to the safety of these structures. With proper tools and techniques, continuous, real-time and automated monitoring systems could evaluate reliability of a structure, identify potential harmful factors and provide assessment for maintenance decisions during its service life. Traditional wired monitoring systems have advantages in acquiring high-fidelity data and robustness, but they still have some drawbacks. Such systems require electronic cables to deliver data sampled by limit sensors to a central device, which usually could be time consuming and expensive for installation and maintenance. In addition, installing these cabled sensors on large civil structures could sometimes interfere with their everyday normal operations. Fortunately, with developments in Micro-Electro-Mechanical System (MEMS) technology, wireless communications and digital electronics, the advent of wireless sensor network (WSN) [3] technology brings new opportunities for structural health monitoring. Compared with wired systems, monitoring systems using WSNs could benefit from their obvious advantages like cable-free and plug and play deployment, which allows engineers to reduce time and cost for installation. Furthermore, these tiny sensor nodes make it possible for dense network deployments, thus allowing better understanding of the monitored structure.

Currently, many SHM systems based on WSNs have been implemented on various civil structures including bridges, tunnels, and tall buildings and so on. Utilizing 64 Micaz nodes with two different accuracy accelerometers that measure ambient vibrations, Kim and Pakzad *et al.* [4,5] implemented an up to 46-hop WSN monitoring system on the Golden Gate Bridge. Jang, Cho and Rice *et al.* [6,7] deployed and evaluated a wireless smart sensor networks monitoring system consisting of 70 Imote2 nodes with custom-designed SHM sensor boards on the Jindo Bridge. Feltrin and Flouri *et al.* [8,9,10] presented an SHM system based on WSNs that addressed challenges for long-term deployments and was used in deployments for monitoring a cable stayed bridge and a timber footbridge. Bischoff *et al.* [11] proposed an event-based monitoring application using eight Tmote Sky nodes with resistance strain gages on a steel railway bridge. Meanwhile, an integrated SHM system for highway bridges based on customized WSN platforms was developed by Hu *et al.* [12] As for tunnels, Bennett *et al.* [13] designed a WSN based underground monitoring system using Micaz nodes with crackmeters and inclinometers, and the SHM system was tested in the Prague Metro and the London Underground. Zonta *et al.* [14] utilized 16 3MATE! nodes with accelerometers, deformation gauges and thermometers to monitor the health status of a medieval tower. Ou *et al.* [15] installed a WSN monitoring system on a tall building called Diwang Tower with eight sensor nodes and comparisons were made with the cable-based system.

In this paper, we implement a wireless monitoring system for nuclear power plants, especially for monitoring changes in crack width on the surface of reactor containment buildings during pressure tests. Customized WSN platforms are developed and the sensors used include crackmeters and thermometers. Issues like route discovery, time synchronization, data collection and duty-cycling schemes are considered in software design. After some simulation tests, full scale deployment is made at the Daya Bay Nuclear Power Station with 30 sensor nodes. The paper is organized as follows: Section 2 presents background and motivation. Section 3 describes hardware selection and assembly for sensor nodes. Software design issues like route discovery, time synchronization, data transfer protocol and wake-up mechanism are discussed in Section 4. Section 5 presents some simulation tests for evaluating the performance of the system. Section 6 describes the field test of the wireless monitoring system. Finally, Section 7 concludes the paper.

## 2. Background and Motivation

The generation of electricity through nuclear energy has many advantages such as lowering greenhouse gas emission, being reliable, and being independent from natural aspects. However, the safety of nuclear power plants are most important, as any slight mistakes could lead to severe disasters. Chernobyl and Fukushima nuclear accidents are just two good examples. Reactor containment building (RCB), as the fourth and final defense layer for a nuclear reactor, is designed to keep radioactive substances from getting into the environment in operational states and in accident conditions like loss of coolant accidents (LOCAs), and protect the nuclear power plant against external natural and human induced events [16]. Then, usually RCBs have to undergo pressure tests to verify that they comply with the highest safety standards. Pressure tests are conducted during commissioning and in-service tests, which are also called initial and periodic tests. They comprise several measurement phases at different pressure levels to verify that the structural behavior of the RCB is consistent with the design and demonstrate that the leak rate of the RCB does not exceed the specified maximum leak rate. Commissioning tests for the RCB are carried out prior to the first criticality of the reactor and in-service tests are usually carried out every ten years. Pressure tests could last as long as two weeks and one very important task during tests is to inspect the surface of the RCB and measure the changes of crack width on the concrete structure in real-time.

Considering that cracks to be concerned about may appear anywhere of the RCB, on the dome or on the perimeter wall, it is impractical to carry out the monitoring task with wired systems. Currently, used battery powered devices that store data locally and gather them after pressure tests also have drawbacks. The monitoring data cannot be obtained by users in real-time for further analysis and users are not informed if some devices stop working during the test, thus leading to incomplete data. The advantages of WSNs and the successful cases of using them to monitor other structures motivate us to introduce this technology for real-time crack width changes monitoring on the surface of RCBs. As far as we know, this is the first time to use wireless sensor networks technology for RCB monitoring. We develop customized platforms and corresponding software. The deployment in Daya Bay Nuclear Power Station shows the effectiveness of the system.

## 3. Hardware Selection and Assembly

Each wireless sensor node in a structural monitoring system is responsible for three primary functions: data acquisition, signal processing and wireless communication. Correspondingly, a sensor node usually consists of a microprocessor, radio transceiver, data storage, batteries and sensor boards. Up to now, many commercial wireless nodes have been used for structural monitoring. In addition, some researchers have also designed their own platforms to carry out monitoring tasks. Table 1 shows the features of typical commercial nodes [4,5,7,8,17,18,19,20] and some customized ones [12,21,22] used in structural monitoring. Based on the experience of past researchers, we design our own nodes with off-the-shelf components. For sensors, crackmeters and thermometers are selected according to monitoring contents. Our monitoring system typically comprises multiple sensor nodes, several cluster nodes and a sink node that is connected to a laptop.

### 3.1. Sensor Selection

**Figure 1:**
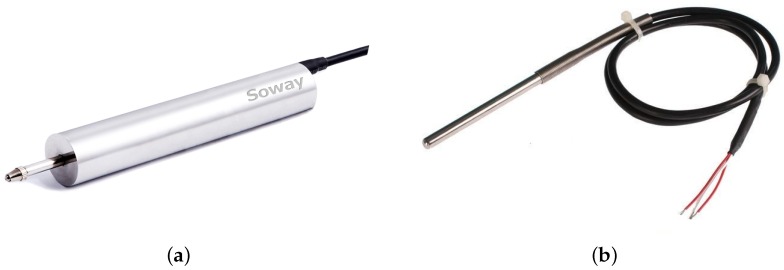
Sensors used for crack width change monitoring. (**a**) LVDT crackmeter; (**b**) PT100 temperature detector.

The most commonly used sensors for structural monitoring are accelerometers, with which the structural vibration response can be captured. In addition, two or three axes, high or low accuracy accelerometers are selected based on specific monitoring requirements. Our monitoring targets are width changes of the cracks on the surface of RCBs, then crackmeters are chosen, and they are based on linear variable differential transformers (LVDTs). LVDTs are displacement transducers and are widely used in industrial applications. They are made up of a central core, a primary coil and a secondary coil with two equal parts that are connected with opposite polarities. The primary coil is supplied with an alternating current, and the two parts of the secondary coil induce different voltages because of different mutual inductances when the core moves between the coils. The difference between the two voltages is the output voltage of the sensor. LVDTs have the advantage of high immunity to electrical noise, and as there is no mechanical contact between the core and coils, LVDTs could increase the expected life. The LVDT (Soway, Shenzhen, China) we choose can be seen in Figure 1a. It has a measurement range from −5 mm to +5 mm with a resolution of 1 *μ*m. The output of the sensor is digital with RS485 interface and the work voltage is 12 Volt direct-current. It also has a built-in temperature compensation mechanism. The precision of the sensor is tested through comparison with the linear comparator, and the result shows that it meets the monitoring requirement. Besides crackmeters, we also use PT100 platinum resistance temperature detectors (Fenghe technology, Wuhan, China) to capture the temperature of the environment for data analysis purposes. The resistance of PT100 remains 100 Ω at 0 ${}^{\circ }$C and changes with temperature. In addition, the temperature information can be obtained through signal converter according to the continuous function relationship between the resistance and temperature. The PT100 platinum resistance temperature detector is shown in Figure 1b.

### 3.2. Node Assembly

**Figure 2:**
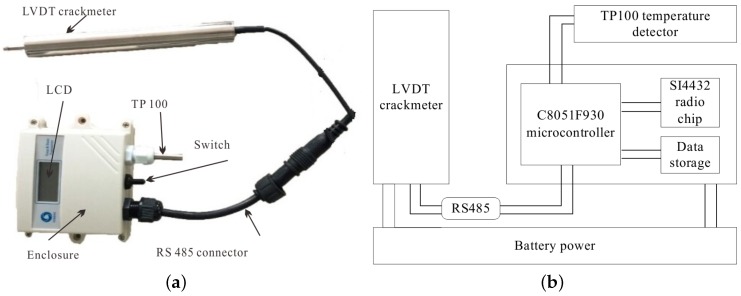
The customized sensor node and its logical structure. (**a**) The sensor node; (**b**) The logical structure of the node.

The components used by nodes mentioned in Table 1 give us a good reference for design customized platforms. In addition, the main components of our customized platforms are the C8051F930 microcontroller and the SI4432 radio chip, both of which are products of Silicon Labs (Austin, TX, US). The 8-bit microcontroller has 4 kB of RAM, 64 kB of on-chip flash memory and maximum 25 MHz clock speed. Its high-speed and low-power features suit the monitoring task well. The reason why the SI4432 is chosen is that low frequency bands are preferred in nuclear power plants. The 433 MHz radio-frequency transceiver offers extremely low receive sensitivity (−121 dBm) coupled with industry leading +20 dBm output power ensures extended range and improved link performance. The advanced radio features of the device reduces overall current consumption and allows the use of the low-cost C8051F930 microcontrollers. The SI4432’s high level of integration also reduces cost while simplifying the nodes design. For nodes measuring width changes of cracks, the LVDT crackmeter and PT100 temperature detector are connected to them. These nodes send commands to sensors, receive feedback from them and then transmit the data wirelessly. In addition, a data storage module for backup in case of data loss in wireless transmission is also attached to the sensor node. It can store up to 5000 data samples, which is enough according to the required sampling period and the duration of pressure tests. A small LCD screen is used for display consideration. As the nodes may carry out monitoring tasks under harsh weather conditions like storm and snow, the hardware components need to be protected for normal operation. Then, a plastic box is used for housing and an external RS485 connector allows the LVDT crackmeter connection, data uploading, battery charging and node reprogramming without opening the enclosure. Figure 2 shows our customized sensor node and its logical structure. There are no sensors connected to cluster nodes in our monitoring system, and the function of cluster nodes is to relay the data transmitted from sensor nodes to the sink node. The only sink node in the system is connected to a laptop through a USB cable. It collects all monitoring data from cluster nodes, or directly from sensor nodes, and stores it in the laptop for further analysis.

### 3.3. Energy Consumption

The sensor nodes are usually battery-powered; therefore, the energy of them is limited, which means that it should be used properly. Energy consumption is one of the most important issues in wireless sensor networks. Every operation of the sensor node affects its life span and the lifetime of the whole monitoring network, respectively. In our system, the sensor nodes are powered by rechargeable lithium batteries with a total capacity of 4000 mAh. In order to make sure that the capacity is large enough to work through pressure test periods, the operation current under idle mode, sampling mode and transmitting mode is measured. We find each sampling state lasts about four seconds and transmitting one packet costs approximately two seconds. According to the typical 30 min sampling period, sensor nodes only sample and transmit twice in one hour. Then, the power consumption under each mode is calculated. Table 2 shows operation current and power consumption of the sensor node in different modes mentioned above. It can be seen from the table that the total power consumption of our sensor node is 2.245 mAh. In addition, the capacity of the batteries ensures a continuous monitoring service life up to 74 days if the initialization stage of the sensor node is not taken into account, which covers the two week pressure test completely.

## 4. Software Implementation

The sensor node in the monitoring system can do nothing without software. Software is the soul of the sensor node to execute sensing, computing, and communication. In addition, the operating system of sensor networks is the cornerstone of software. Taking the features of WSNs into account, many embedded operating systems special for sensor networks like TinyOS, SOS, MANTIS OS and Contiki have been developed by researchers. TinyOS [23,24] is an open source, lightweight operating system developed by UC Berkeley for low-power wireless sensor nodes. It provides a set of important services and abstractions, making it easier to build sensor network applications. Applications and the system itself are written in a C dialect, called nesC language. It has been run on many generic platforms and used in a wide variety of monitoring applications for WSNs [7,12,25]. SOS [26] is an operating system for mote-class WSNs developed by the NESL at University of California (Los Angeles, CA, USA). It uses a common kernel to implement basic services. In addition, the prominent characteristic of SOS is its dynamic reconfigurability, which allows dynamic addition, modification, and removal of network services. However, currently, SOS is no longer under active development. MANTIS OS [27] is an open source, multi-threaded operating system written in C for wireless sensor networking platforms. It supports many platforms and has been used in several applications. Contiki [28] is a tiny, highly portable operating system for small devices developed by a world-wide team of developers. It is a very promising operating system as it is easy to connect to the Internet. We have not currently introduced any operating systems into our monitoring system, and we just utilize C language to implement the software functions. In initialization stage, route discovery and time synchronization of nodes are considered, and we focus on transmitting protocol and duty cycling in monitoring stage. The graphic user interface (GUI) at the sink node side is also mentioned.

### 4.1. Initialization Stage

In the initialization stage, nodes need to prepare for data collection, and there are two main things they should complete during this stage: one is route discovery and the other is time synchronization. Route discovery is very important as it is the precondition of data collection. In our monitoring system, we implement a hierarchy routing protocol similar to LEACH protocol [29]. The difference is that there is no cluster heads selection phase because the sink nodes and cluster nodes in our system are existing cluster heads. First, the sink node is assigned a level 0 and broadcasts a route discovery message containing its identity and level. All of the cluster nodes and sensor nodes receiving this message set the sink node as their parent and assign themselves a level 1. Then, the cluster nodes in level 1 broadcast a new a route discovery message, and this process continues until all nodes in the network assign a level. Nodes that already have a parent will ignore later broadcast messages. In this way, a routing tree rooted at the sink node is established and all the sensor nodes send data directly to the sink node or through cluster nodes in the network. Time synchronization is one of the fundamental issues in WSNs. All nodes in the monitoring network sharing a common timescale makes the measured data meaningful. Some scheduling protocols like time division multiple access (TDMA) require time synchronization so that transmissions do not interfere. In addition, an accurate time is essential for efficient duty cycling and helps save energy. The required synchronization accuracy is not high in our monitoring system as the typical sampling period is 30 min. In addition, the algorithm we achieved in our system is a simplified flooding time synchronization protocol (FTSP) [30]. The synchronization process is conducted between neighbor levels of the spanning-tree established in route discovery phase. Each cluster head broadcasts a message time-stamped at the media access control (MAC) layer, and child nodes of that cluster head record the corresponding time from their respective local clocks at message reception. Only clocks offset between them are estimated, and, once synchronized, the network can keep the synchronization error in the second range during the whole monitoring period.

### 4.2. Monitoring Stage

**Figure 3:**
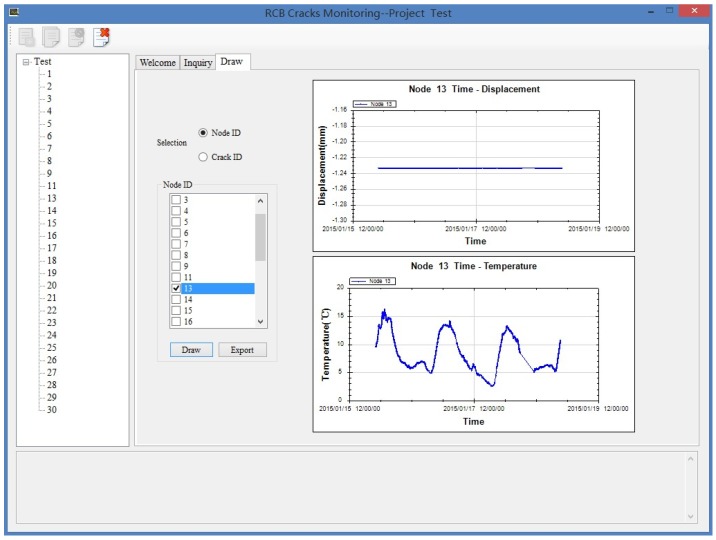
Graphical user interface (GUI) of the monitoring system.

The main task for sensor nodes in the monitoring stage is to measure changes of crack width and send them to cluster heads. As the widths of concerned cracks change slowly during pressure tests, a 30 min sampling period is enough for capturing the variation of cracks. In addition, duty cycling schemes are employed by sensor nodes. Every 30 min, sensor nodes wake up to get data from LVDT crackmeters and PT100 temperature detectors, store it and send it to cluster heads. Then, they go into idle mode and turn off their sensors and radios. In this way, sensor nodes can save a lot of energy, as they sleep most of the time. Although cluster heads do not need to monitor the cracks, they may need to always keep their radio on to receive packets from sensor nodes. Then cluster heads may consume much more power than sensor nodes, and they need to be powered by batteries with more capacity or use alternating current (AC) power when possible. Thanks to the time synchronization scheme, TDMA is used by sensor nodes for media access control to prevent transmission interference. Sensor nodes turn off their radio after sending messages out in order to save energy, which means usually they do not receive messages from cluster heads during the monitoring stage. Then, an unreliable data transfer protocol is used to adapt this feature. Sensor nodes just send measured data once to cluster heads, and there is no feedback from cluster heads about packet loss and thus no retransmission occurs. Data loss is inevitable in wireless transmission, and tests in Section 5 about data loss rate of our system under different sampling periods show that data loss rate under typical 30 min sampling period is tolerable. In addition, this means that the unreliable data transfer protocol is appropriate for our system. Unlike other high sampling rate applications, the amount of data in our system is low because of low sampling periods, and sensor nodes just send what they measure without any compression. The GUI that runs on the laptop receives all the monitoring data of the network with the help of the sink node, and it also provides functions for storing, inquiring and drawing data. The GUI is shown in Figure 3, which is implemented with C# language.

## 5. Wireless Transmission Tests

When the wireless monitoring system is implemented, transmission tests are carried out before full scale deployment to find out the transmission range of sensor nodes and the data loss rate of the system.

The purpose of transmission range test is to determine how far the radio we choose can transmit at most in the open air and guide the deployment of cluster nodes. Considering the structure of RCB and the place where the sink node has to be put, both horizontal and vertical transmission range tests are carried out. In horizontal tests, the sink node is connected to the laptop for receiving messages from sensor nodes, and one sensor node is placed near the sink node to send messages as references. Three other sensor nodes are placed at a horizontal distance of 30 m from the sink node to send messages, and then at the distance of 50 m, 70 m, 100 m, 120 m and 150 m, respectively. The transmission power levels of these sensor nodes are kept the same during the test, the sampling period is 1 min, and three sensor nodes are placed at each distance for 5 min. Test results show that the sink node can receive all messages when sensor nodes are placed at the distance of 30 m, 50 m, 70 m and 100 m, and it cannot receive all the messages at the distance of 120 m, while no messages are received at a distance of 150 m except those from the reference sensor node. From this test, we know that our sensor nodes ensure a horizontal transmission distance of 100 m. Most sensor nodes will be deployed at the dome area of the RCB, but the sink node needs to be placed on the ground, which is at a vertical distance of 50 m from the dome due to space and power supply constraints. Then, a vertical transmission range test is conducted on a 20-story building. Similar to the horizontal test, the sink node and reference sensor node are placed on the ground, three sensor nodes are placed at level 5 with line of sight, then at level 10 and 15. The sampling period and duration at each level are the same as the horizontal test. Results show that even when sensor nodes are placed at level 15, a vertical distance of about 50 m, the sink node on the ground can receive all the messages from them, which means that it is feasible to put the sink node on the ground for convenience.

**Figure 4:**
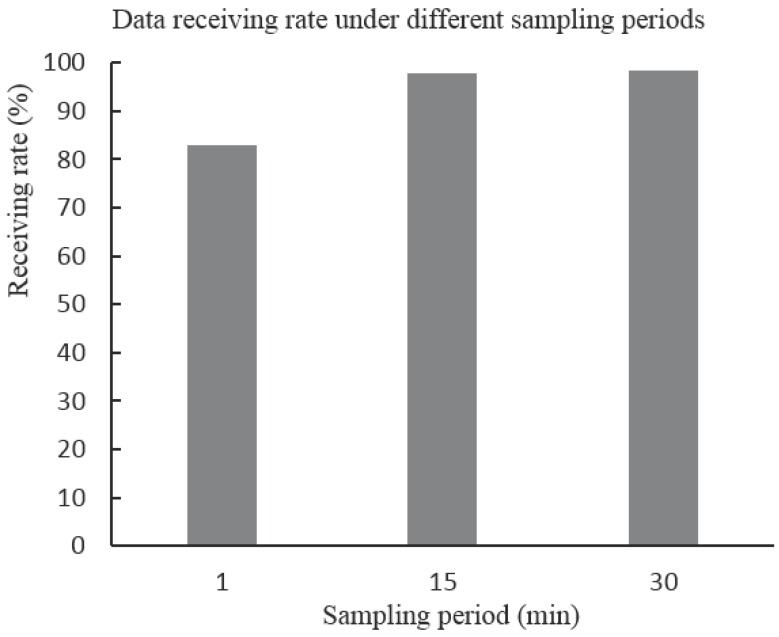
Average data receiving rate under different sampling periods.

As mentioned in Section 4, an unreliable data transfer protocol is used for saving energy consideration, and this may cause data loss during wireless transmission due to interference and other environment factors. Then, a data loss rate test is carried out to evaluate the reliability of the wireless monitoring system. Thirty sensor nodes are casually deployed on the top floor of our office building and several LVDT sensors are installed on the surface of wall cracks. The sampling period is set at 1 min, then 15 and 30 min, respectively. All sensor nodes get and send data to the sink node under different sampling periods, and each test lasts 24 h for each sampling period. In addition, data loss rate is calculated based on the sampling period and the messages received at the sink node. Figure 4 shows the average data receiving rate under different sampling periods. It can be seen from the figure that about 83% of data is received at the sink node under the 1 min sampling period during the 24 h test. In addition, the data loss rate under 15 and 30 min sampling periods is relatively low compared with that of the 1 min sampling period. Considering that the typical sampling period for our system is 30 min, the data loss rate is tolerable and the unreliable data transfer protocol meets the demand of our system and saves energy at the same time.

## 6. Real-Life Deployment

Daya Bay Nuclear Power Station is located in Guangdong province, which is the first commercial nuclear power station in mainland China. As one of the earliest and largest joint venture projects launched under China’s Open Door Policy, it began to be built in 1987 and was completed in 1993, and French and British technology were used during the construction. The plant has two pressurized water reactor generating units with a total electrical capacity of 1968 MW. It produces around 14 billion kWh of electricity per year, and the primary consumers of this nuclear power station are Hong Kong and Guangdong province, consuming 70% and 30%, respectively. Daya Bay Nuclear Power Station is under strict management and operation to maintain safety. Our full scale deployment is carried out during its every ten years’ in-service pressure test.

**Figure 5:**
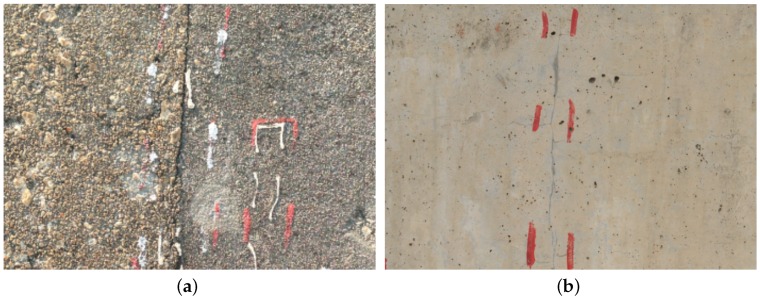
The geometry of cracks on the surface of RCB. (**a**) Crack shape one; (**b**) Crack shape two.

The pressure test is conducted to check the integrity and leak rate of the concrete containment building that houses the nuclear reactor. In addition, one important task during the test is to inspect the surface of the RCB and measure the changes of crack width on the concrete structure. Before the pressure test, a manual inspection is conducted to find out cracks that need to be monitored on the surface of the RCB. Then, the locations of concerned cracks are reported and the deployment of sensor nodes is based on the report. Figure 5 shows the geometry of concerned cracks and they may appear on the dome or on the perimeter wall of the RCB.

**Figure 6:**
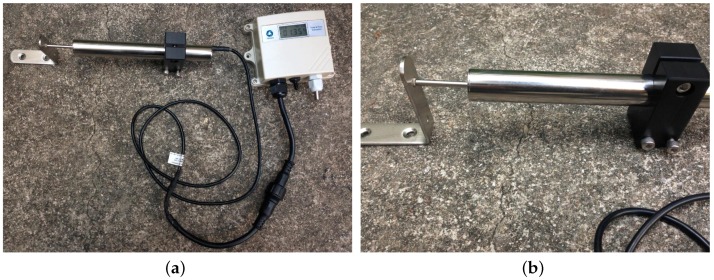
The installation of LVDT crackmeters. (**a**) The sensor node; (**b**) Installation details diagram.

Sensor nodes with LVDT crackmeters are installed on the surface of concerned cracks. The details of installation are illustrated in Figure 6. First, the LVDT base is fixed on one side of the crack with plastic clamps, making the measurement direction of the sensor vertical to the crack’s direction. Then, the retractable part of the LVDT is fixed on the other side of the crack using a mounting bracket according to the range of the sensor. As we mainly focus on the changes of cracks’ width during the pressure test, the initial width of cracks are not measured.

**Figure 7:**
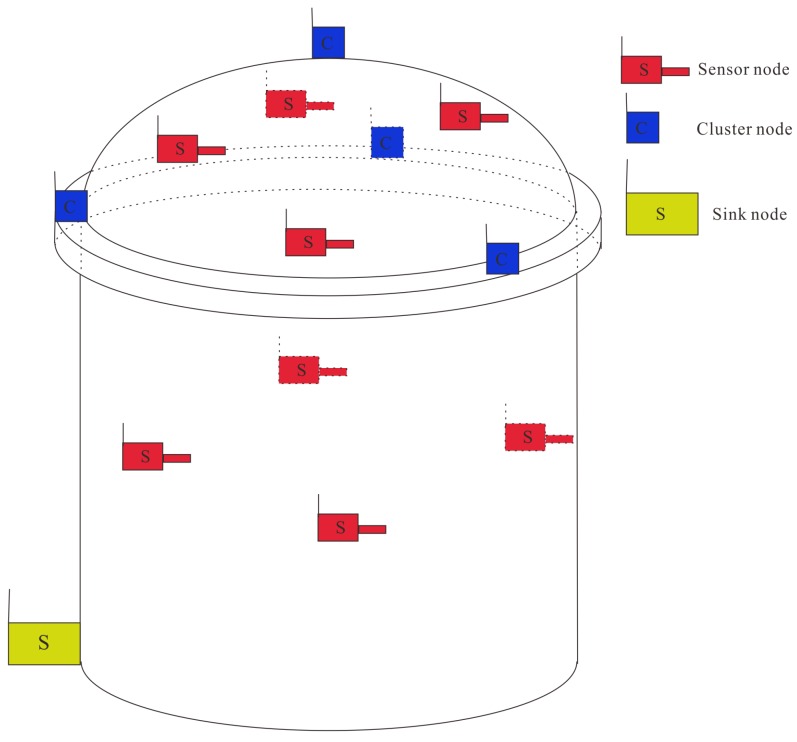
Schematic layout of the wireless monitoring system in the RCB.

The quantity and function of nodes used in the full scale deployment is shown in Table 3. Thirty sensor nodes are installed on the surface of concerned cracks according to the results of manual inspection. Considering the transmission range of sensor nodes and the structure of the containment building, four cluster nodes are used for data relay purpose in the wireless monitoring system. They are evenly deployed on the top and around the dome area. The only sink node responsible for data collection is connected to the laptop and placed on the ground for continuous power supply. Figure 7 shows the schematic layout of the wireless monitoring system on the RCB.

**Figure 8:**
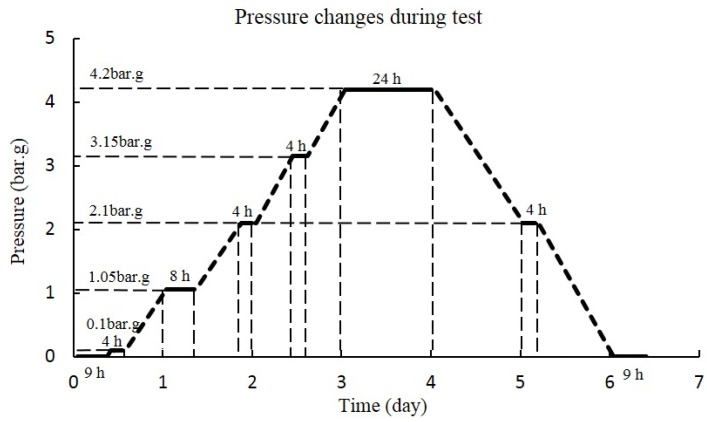
Pressure changes during the pressure test.

When the deployment of the wireless monitoring system is completed, the pressure test starts. The reactor is shut down and compressed air is pumped into the containment building, the building is pressurized to predetermined pressure under several measurement phases at different pressure levels, which can be seen in Figure 8. At first, the pressure stays at 0 bar (gage) for 9 h, then it goes up to a certain pressure and stays there for some time. In addition, the maximum pressure is 4.2 bar (gage), which lasts for 24 h. After that, the pressure decreases to 0 bar (gage) gradually. The whole pressure test process lasts about seven days.

Just before the pressure test, the wireless monitoring system runs route discovery and time synchronization algorithms to prepare for data collection. Then, the monitoring stage starts along with the pressure test, and sensor nodes record the changes of cracks’ width due to thermal and pressure impacts and send them to cluster heads every 30 min. Cluster nodes help sensor nodes relay data to the sink node. The sink node collects, stores and displays all the data in real-time. The monitoring stage lasts seven days until the pressure test stops. The sink node can acquire data from all of the 30 sensor nodes, and the average data receiving rate is similar to the transmission test that we have done before, which verifies the efficiency of the wireless monitoring system. In addition, only about 10% of the energy is consumed on average, which is consistent with our previous theoretical calculations.

## 7. Conclusions

The advent of wireless sensor networks brings both opportunities and challenges for structural health monitoring. We enjoy the convenience of wireless transmission but suffer limited resources at the same time. In this paper, we present a real-time monitoring system for cracks on the surface of reactor containment buildings during pressure tests based on wireless sensor networks. Sensors like crackmeters and temperature detectors are chosen and customized platforms are designed and implemented to meet the monitoring requirements. Meanwhile, software protocols like route discovery, time synchronization and data transfer are developed to take the limited resources into account. A graphical user interface is programmed to receive, store and display data. The monitoring system is evaluated with several experimental tests before real-life deployment. In addition, the full scale deployment of the system at Daya Bay Nuclear Power Station with 30 sensor nodes is quite successful. Despite the successful deployment, there are still some improvements that can be made for our system. Currently only crackmeters and temperature detectors are used in our nodes, and more sensors may be added for multiple purposes in the future. The topological structure of the monitoring network is established at the initialization stage and is static; thus, it is not robust to failures, especially for cluster head errors. Therefore, dynamic topology will be a better choice for the system. The system is mainly designed for low sampling frequency tasks as tailored time synchronization protocols are developed. However, it may need to be redesigned to suit some high sampling frequency monitoring projects. Similarly, the unreliable data transfer protocol only works well with low sampling frequency, reliable protocols or data recovery mechanisms that are required for high sampling frequency tasks. We believe our system can be easily adapted to many monitoring tasks if these improvements are made.

## Figures and Tables

**Table 1:** Typical nodes used in structural monitoring.

Node	Microprocessor	Radio Chip	Data Storage (Bytes)	Batteries
Mica2	ATmega128L	CC1000	512 K	2 × AA
MicaZ	ATmega128L	CC2420	512 K	2 × AA
Imote2	XScalePXA271	CC2420	32 M	3 × AAA
Tmote-sky	MSP430F1611	CC2420	1 M	2 × AA
S-mote	MSP430F1611	CC2420	1 M	2 × AA lithium
WiSSM	ATmega128	Maxstream 9XCite	128 K	5 × AA

**Table 2:** Operation current and power consumption in different modes.

Operation Mode	Operation Current	Power Consumption
Idle	2 mA	2 mAh
Data sampling	80 mA	0.178 mAh
Data transmitting	60 mA	0.067 mAh

**Table 3:** Quantity and function of nodes used in the full scale deployment.

Node Types	Quantity	Function
Sensor node	30	Data sampling and transmission
Cluster node	4	Data relay
Sink node	1	Data collection
